# Measuring drug similarity using drug–drug interactions

**DOI:** 10.1002/qub2.38

**Published:** 2024-03-31

**Authors:** Ji Lv, Guixia Liu, Yuan Ju, Houhou Huang, Ying Sun

**Affiliations:** ^1^ College of Computer Science and Technology Jilin University Changchun China; ^2^ Key Laboratory of Symbolic Computation and Knowledge Engineering of Ministry of Education Jilin University Changchun China; ^3^ Sichuan University Library Sichuan University Chengdu China; ^4^ College of Chemistry Jilin University Changchun China; ^5^ Department of Respiratory Medicine The First Hospital of Jilin University Changchun China

**Keywords:** drug similarity, drug–drug interactions, drug combinations, synergy effect, clustering, semi‐supervised learning

## Abstract

Combination therapy is a promising approach to address the challenge of antimicrobial resistance, and computational models have been proposed for predicting drug–drug interactions. Most existing models rely on drug similarity measures based on characteristics such as chemical structure and the mechanism of action. In this study, we focus on the network structure itself and propose a drug similarity measure based on drug–drug interaction networks. We explore the potential applications of this measure by combining it with unsupervised learning and semi‐supervised learning approaches. In unsupervised learning, drugs can be grouped based on their interactions, leading to almost monochromatic group–group interactions. In addition, drugs within the same group tend to have similar mechanisms of action (MoA). In semi‐supervised learning, the similarity measure can be utilized to construct affinity matrices, enabling the prediction of unknown drug–drug interactions. Our method exceeds existing approaches in terms of performance. Overall, our experiments demonstrate the effectiveness and practicability of the proposed similarity measure. On the one hand, when combined with clustering algorithms, it can be used for functional annotation of compounds with unknown MoA. On the other hand, when combined with semi‐supervised graph learning, it enables the prediction of unknown drug–drug interactions.

## 1 INTRODUCTION

Antimicrobial resistance is a growing public health concern [1, 2], and combination therapy (i.e., antibiotic combinations) is a promising strategy to combat antibiotic resistance [3, 4, 5]. To date, a great number of computational models have been developed to predict antibiotic combinations [6, 7, 8]. Most of these models are based on the similarity assumption (i.e., similar drugs have similar drug–drug interactions (DDIs)) [9, 10, 11, 12, 13, 14, 15]. Previous studies have shown that drugs can be classified into specific groups based on their mechanisms of action (MoA), chemical structure, or physicochemical properties, and the type of drug–drug interactions is often related to these groups [13, 16, 17, 18, 19]. Based on drug similarity, it is possible to generate a list of drugs that are similar to a given drug. This approach allows for functional annotation of compounds with unknown MoA and the prediction of unknown drug–drug interactions.

Drug similarity is a fundamental concept in drug discovery [20], and various approaches have been developed to quantify drug similarity [20, 21]. For example, drugs can be represented as 166 bit MACCS keys, where each bit corresponds to a particular substructure, and the Tanimoto coefficient can be used to calculate the similarity between drugs [22]. Lv et al. proposed a network proximity method combined with network propagation to assess the relationship between drug targets [11], revealing that drug pairs with negative network proximity exhibit pharmacological similarity. Furthermore, drugs can be classified into 14 main groups based on the anatomical therapeutic chemical (ATC) codes [23], and the Jaccard coefficient can be used to measure ATC similarity [10]. These methods primarily rely on drug characteristics. However, in this study, we specifically focus on the DDI network structure itself.

**FIGURE 1:**
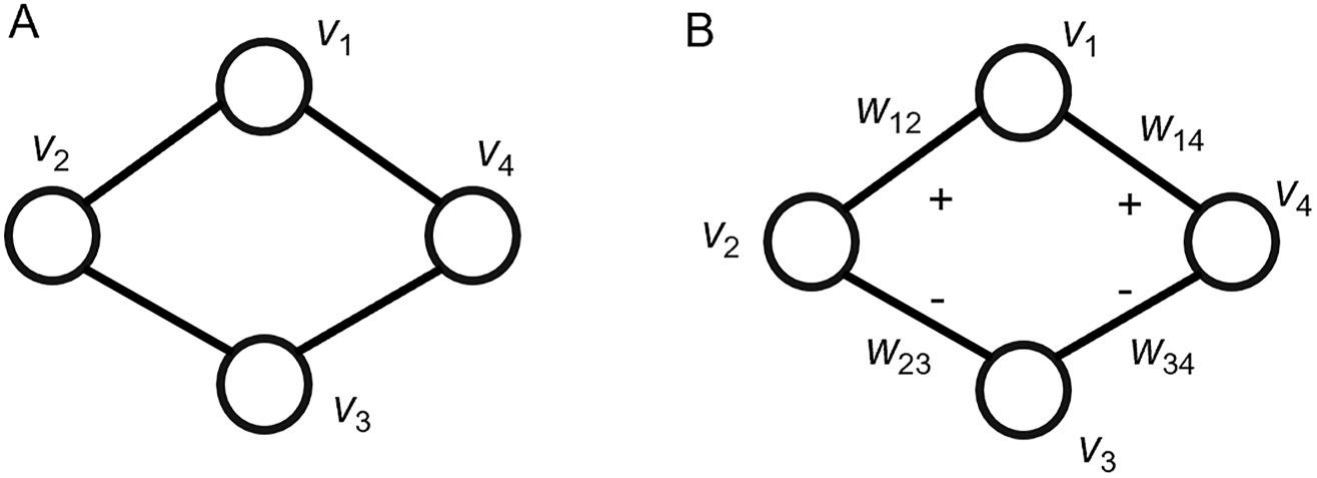
Example of (A) an unsigned network and (B) a signed network.

In the case of an unsigned network, nodes that share many neighbor nodes are considered similar. In Figure 1A, *v*_1_ and *v*_3_ share two neighbor nodes (*v*_2_ and *v*_4_), indicating structural equivalence between the two nodes. However, in a DDI network (i.e., signed network), drug pairs can be either synergistic or antagonistic combinations [24]. As shown in Figure 1B, *v*_1_ and *v*_3_ also have two common neighbor nodes (*v*_2_ and *v*_4_), but the symbol (edge type) of *w*_12_ (*w*_14_) is different from *w*_23_ (*w*_34_). Therefore, *v*_1_ and *v*_3_ are not considered similar in the signed network. To address this disparity, we proposed a similarity measure specifically designed to assess drug similarity in DDI networks.

## 2 RESULTS AND DISCUSSION

Next, we will demonstrate the potential applications of the drug similarity measure.

### 2.1 Clustering DDI network

Community structure, which refers to the division of nodes into groups based on their connectivity, characteristics, or roles in the network, is a prominent feature of complex networks [25].

**FIGURE 2:**
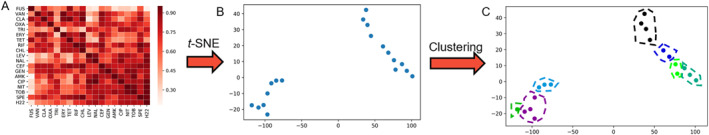
Clustering drug–drug interaction network. (A) Drug similarity matrix. (B) *t*‐SNE analysis. (C) Hierarchical clustering.

**FIGURE 3:**
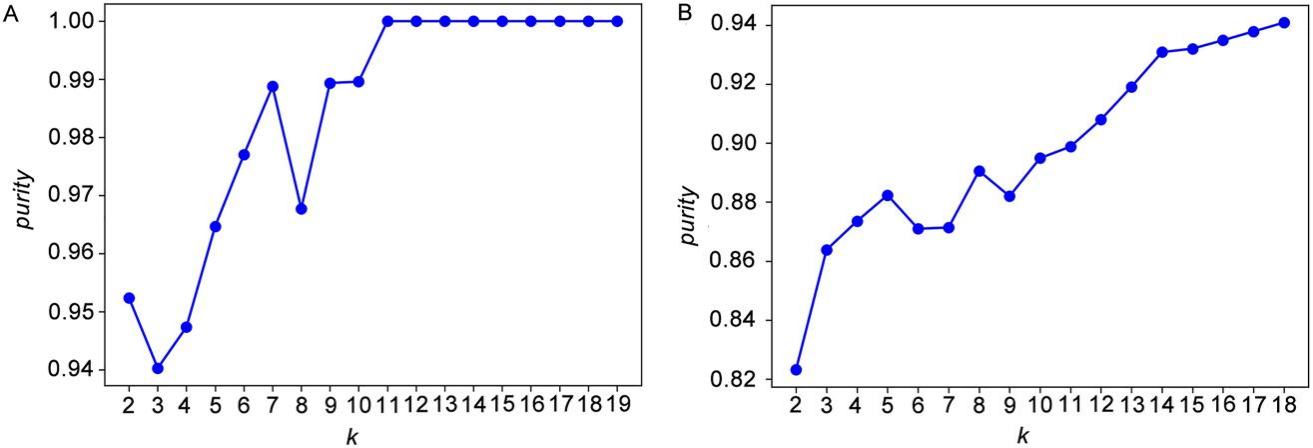
The relation between the number of clusters (*k*) and *purity*. (A) *E*. *coli* MG1655 dataset. (B) *E*. *coli* BW25113 dataset.

In this study, our objective was to cluster drugs with similar drug–drug interactions. To achieve this, we first calculated the node similarity (Equation 6) of each drug pair, resulting in a 19 × 19 similarity matrix (Figure 2A) and a 68 × 68 similarity matrix. These nodes’ (drugs’) similarity matrices were then utilized for clustering algorithms, such as spectral clustering and hierarchical clustering. To determine the number of clusters, we examined the relationship between the number of clusters (*k*) and *purity* (Equation 7). As shown in Figure 3, satisfying results were obtained for the *Escherichia coli* MG1655 and *E*. *coli* BW25113 datasets when *k* = 7 and *k* = 8, respectively. Consequently, the DDI networks of the *E*. *coli* MG1655 and *E*. *coli* BW25113 datasets were clustered into 7 and 8 groups, respectively. Subsequently, we compared the clustering performance of various algorithms. As shown in Table 1, a combination of *t*‐SNE and hierarchical clustering showed the best clustering performance. It is worth noting that drug combination datasets often contain noise [26]. Clustering the raw similarity matrices can lead to excessive noise and unnecessary computations. To mitigate this issue, we employed *t*‐SNE to compress the similarity matrices before performing clustering to help reduce the effect of noise.

**TABLE 1:** Clustering performance using different clustering algorithms.

	*E*. *coli* MG1655	*E*. *coli* BW25113
Spectral clustering	0.957	0.840
Hierarchical clustering	0.953	0.879
*t‐*SNE + hierarchical clustering	0.989	0.891

Finally, we compared the performance of clustering using node similarity (Equation 6) and existing similarity measures (e.g., structural similarity, MoA similarity). As expected, clustering using node similarity demonstrated superior performance (Table 2 and Figures S1,S2). Given that our method is based on the network structure itself, it can be regarded as a highly effective approach for DDI network clustering, often considered as the ‘holy grail’ method in this context.

**TABLE 2:** Clustering performance using different similarity measures.

	*E*. *coli* MG1655	*E*. *coli* BW25113
Structural similarity	0.914	0.714
MoA similarity	0.886	0.700
Node similarity (our method)	0.989	0.891

**FIGURE 4:**
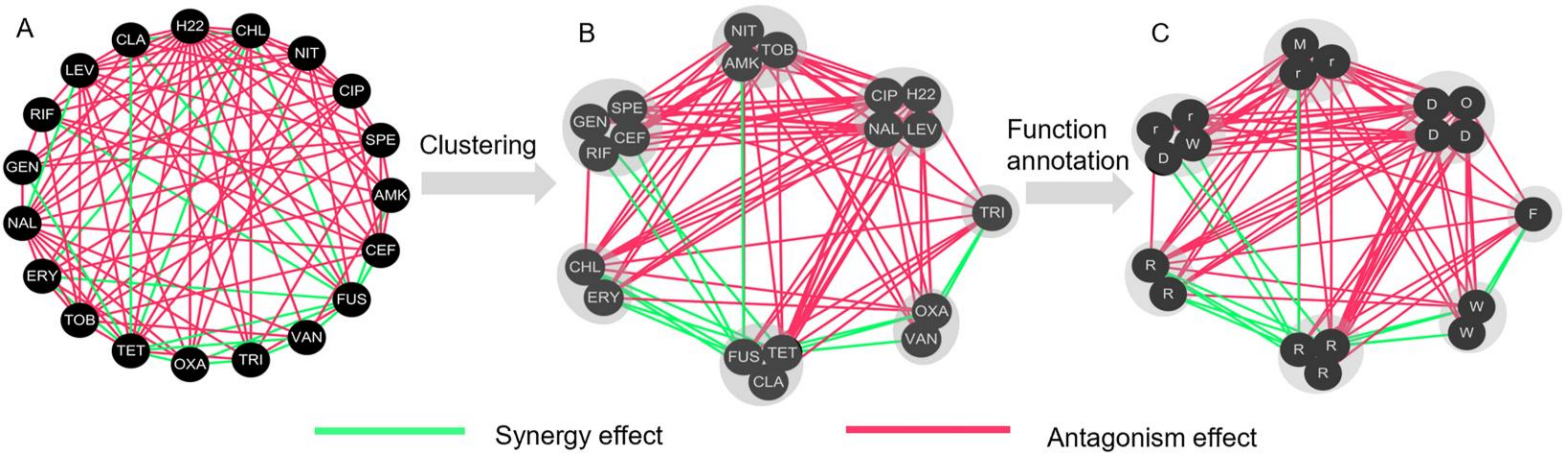
(A) The drug–drug interaction (DDI) network of *E*. *coli* MG1655. (B) The clustered DDI network. (C) Function annotation for the clustered DDI network. r, 30S protein synthesis inhibitor; R, 50S protein synthesis inhibitor; F, folic acid synthesis inhibitor; M, multiple mechanisms; W, cell wall synthesis inhibitor; and O, oxidizing agent. Synergistic and antagonistic drug combinations are colored in green and red, respectively.

Taking *E*. *coli* MG1655 as an example (Figure 4A), we divided the drugs into 7 groups. In general, the group–group interactions are monochromatic (Figure 4B).

Drug similarity can be assessed based on structural similarity or MoA similarity [21]. We performed correlation analyses to investigate the relationship between these similarity measures and node similarity. The correlation coefficient between structural similarity (Tanimoto similarity, molecular fingerprints: MACCS keys) and node similarity was 0.02 (*p*‐value = 0.84), while the correlation coefficient between MoA and node similarity was 0.37 (*p*‐value <0.01). This indicates that our method reflects MoA similarity rather than structural similarity. For instance, consider the drugs AMK, GEN, and TOB, which are all aminoglycoside antibiotics and possess highly similar chemical structures (T_GEN‐AMK_ = 0.716 and T_GEN‐TOB_ = 0.719, Tanimoto similarity). Aminoglycosides bind to position H44 on the 30S ribosomal subunit [27]. However, GEN also has a second binding site (H69) on the 50S ribosomal subunit [28]. This distinct binding site leads to different interactions between these drugs with TET. The combination of GEN with TET is synergetic because drug pairs targeting different binding sites on the same biological processes can bypass redundant mechanisms [11]. Interestingly, these drug groups are associated with the MoA of drugs (Figure 4C). Similarly, the drugs VAN and OXA, which are inhibitors of cell wall synthesis, cluster together. Drugs targeting DNA (e.g., CIP, LEV, and NAL) also cluster together. However, drugs affecting protein synthesis do not form a specific class but can be further classified as bactericidal or bacteriostatic drugs [29]. For example, AMK, TOB, and TET are 30S ribosomal subunit inhibitors. However, AMK and TOB are bactericidal drugs, while TET is a bacteriostatic drug. This distinction leads to different interactions between these drugs and cell wall synthesis inhibitors (e.g., VAN and OXA). Specifically, combinations of TET with cell wall synthesis inhibitors are synergistic, while combinations of AMK (or TOB) with cell wall synthesis inhibitors are antagonistic. This highlights the challenge of obtaining meaningful clustering results using single drug information alone [13]. Another potential application of the drug similarity measure is the functional annotation of compounds with unknown MoA. To illustrate this, we tested hydrogen peroxide (H_2_O_2_) as a compound with an unknown MoA. Interestingly, we observed that hydrogen peroxide and drugs targeting DNA were grouped together, implying that hydrogen peroxide may share a similar MoA with drugs that target DNA. In fact, there is evidence that hydrogen peroxide can react with the intracellular pool of unincorporated iron, leading to DNA damage [30].

### 2.2 Prediction of unknown drug–drug interactions

In the microbiology laboratory, synergy or antagonism can be determined using the checkerboard method [3, 4]. However, this approach is both time‐consuming and labor‐intensive, making it unsuitable for high‐throughput screening. According to the concept of clustering, where similar drugs tend to exhibit similar interactions (Figure 4B), we can classify drug combinations using graph semi‐supervised learning. For example, if drug A and drug B are synergistic and drug A is similar to drug C, there is a possibility that drug A and drug C may also form a synergistic drug combination.

**FIGURE 5:**
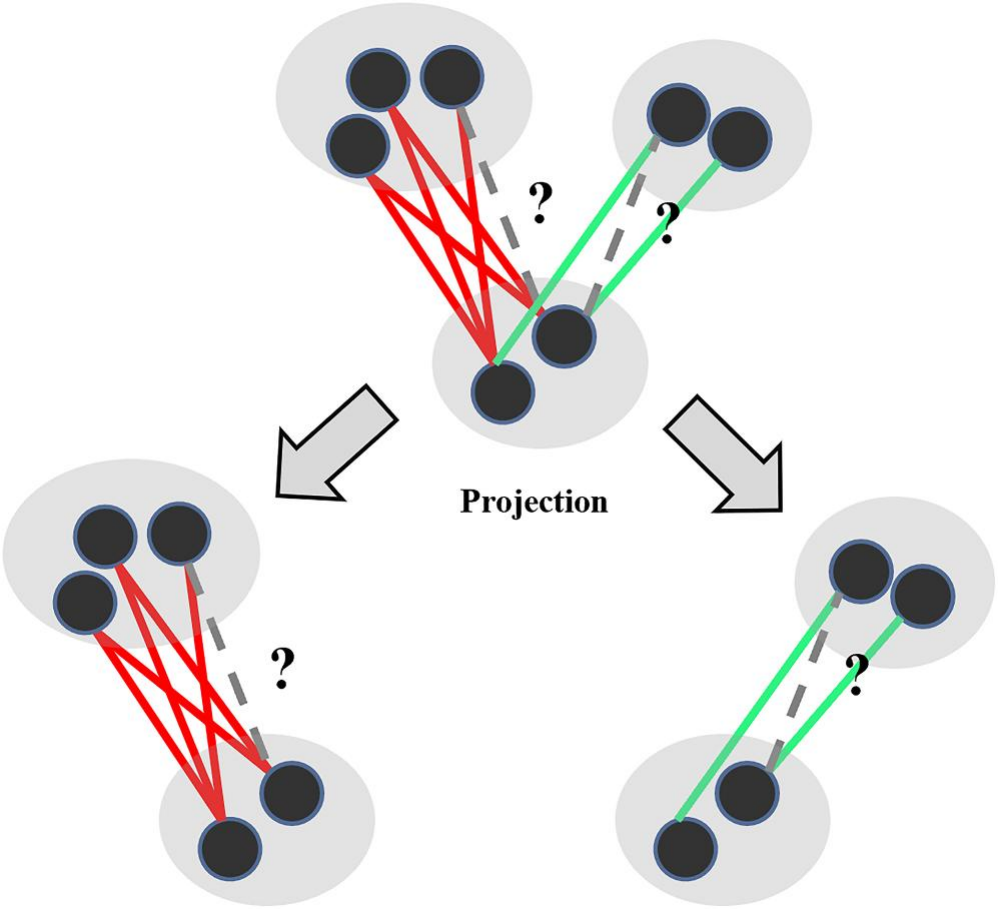
A diagram of network projection. Synergistic and antagonistic drug–drug interactions are colored in green and red, respectively.

Existing drug combination prediction methods based on graph semi‐supervised learning have traditionally relied on drug properties (e.g., chemical structure, MoA) [9, 10, 11, 31]. However, obtaining certain properties (e.g., MoA) can be challenging due to limited availability. In order to address this issue, we calculated drug similarities using known drug–drug interactions (Equation 6) and then used semi‐supervised learning to predict unknown drug interactions (Equations 15 and 16). Specifically, we first utilized a network projection method to project the network into two subnetworks (Figure 5). Subsequently, we applied semi‐supervised learning on each of the two subgraphs and determined the type of drug interactions by comparing the scores of the two subgraphs. The complete predicted results are listed in Tables S1,S2. To evaluate the performance of our method, we employed *precision* (Equation 17), *recall* (Equation 18), *accuracy* (Equations 19), and *F*1‐value (Equation 20). In comparison to existing similarity measures (e.g., structural similarity, MoA similarity), our method demonstrated superior performance (Tables 3 and 4). The complete set of predicted results using structural similarity or MoA similarity are listed in Tables S3–S6. Significantly, our method does not rely on additional information (e.g., chemical structure and MoA) beyond the known drug–drug interactions.

**TABLE 3:** Performance comparison of our method and existing similarity measures for the *E*. *coli* MG1655 dataset.

	*Precision*	*Recall*	*Accuracy*	*F*1
Structural similarity	1	0.50	0.90	0.67
MoA similarity	0.67	0.50	0.86	0.57
Node similarity (our method)	1	0.75	0.95	0.86

**TABLE 4:** Performance comparison of our method and existing similarity measures for the *E*. *coli* BW25113 dataset.

	*Precision*	*Recall*	*Accuracy*	*F*1
Structural similarity	0.88	0.64	0.81	0.74
MoA similarity	1	0.48	0.78	0.65
Node similarity (our method)	0.88	0.64	0.81	0.74

## 3 CONCLUSIONS

In this study, we proposed a practical drug similarity measure based on the DDI network itself. The effectiveness of the drug similarity measure was demonstrated through experiments on the DDI network. To summarize, our proposed method allows for the functional annotation of compounds with unknown MoA using the clustering method. Additionally, we can predict unknown drug‐drug interactions using semi‐supervised graph learning.

## 4 MATERIALS AND METHODS

### 4.1 Dataset

We used recent experimental data of *E*. *coli* MG1655 [26] and *E*. *coli* BW25113 [18]. For *E*. *coli* MG1655, the dataset consisted of 18 antibiotics and an oxidizing agent (Table 5) covering various drug classes (e.g., protein synthesis, DNA gyrase, and cell wall). The interaction types of drug pairs (171 drug pairs, Table S7) were measured using the *α*‐score [24]. For *E*. *coli* BW25113, the dataset (Table S8) consisted of 40 antibiotics, 5 food additives, 11 human‐targeted drugs, etc. The interaction types of drug pairs (1079 drug pairs, Table S9) were measured using the Bliss score [32].

**TABLE 5:** Drugs listed in the *E. coli* MG1655 dataset and their abbreviation and the mechanism of action.

Drugs	Abbreviation	Mechanism of action	Phenotype
Amikacin	AMK	Protein synthesis, 30S	Bactericidal
Gentamicin	GEN	Protein synthesis, 30S	Bactericidal
Tobramycin	TOB	Protein synthesis, 30S	Bactericidal
Tetracycline	TET	Protein synthesis, 30S	Bacteriostatic
Chloramphenicol	CHL	Protein synthesis, 50S	Bacteriostatic
Clarithromycin	CLA	Protein synthesis, 50S	Bacteriostatic
Erythromycin	ERY	Protein synthesis, 50S	Bacteriostatic
Ciprofloxacin	CIP	DNA gyrase	Bactericidal
Levofloxacin	LEV	DNA gyrase	Bactericidal
Nalidixic acid	NAL	DNA gyrase	Bactericidal
Trimethoprim	TRI	Folic acid biosynthesis	Bacteriostatic
Oxacillin	OXA	Cell wall	Bactericidal
Cefoxitin	CEF	Cell wall	Bactericidal
Hydrogen peroxide	H22	Oxidative stress	Bactericidal
Nitrofurantoin	NIT	Multiple mechanisms	Bactericidal
Fusidic acid	FUS	Elongation factor—protein synthesis	Bacteriostatic
Rifampicin	RIF	RNA synthesis	Bactericidal
Vancomycin	VAN	Cell wall	Bactericidal
Spectinomycin	SPE	Protein synthesis, 30S	Bacteriostatic

### 4.2 Measuring drug similarity in the DDI network

#### 4.2.1 DDI network

In a DDI network (*G*), which can be considered a typical signed network, the nodes represent drugs (*V*), and the edges represent drug–drug interactions (*W*) [33]. For pairwise drug combinations [24], if the combined effects are greater than, equal to, or less than the sum of the individual drugs, then drug combinations are synergistic, additive, or antagonistic, respectively.

*G* can be represented by an adjacency matrix **A**. If *G* has *n* nodes, then the adjacency matrix **A** is an *n* × *n* square matrix and is defined as follows:

(1) $$A_{ij}=\left\{\begin{array}{l} +1,\;\text{synergy}\;\text{effect}\;\\ 0,\text{additive}\;\text{effect}\;\\ -1,\;\text{antagonism}\;\text{effect}\; \end{array}\right.$$

The adjacency matrix has two identical characteristics: Equation (1) their diagonal elements are 0, and Equation (2) they are symmetric.

#### 4.2.2 Node similarity

For a DDI network, neighbor nodes can be positive neighbor nodes *N* ^*+*^ or negative neighbor nodes *N* ^*−*^. Figure 1B shows a toy signed network. Considering *v*_1_ and *v*_3_, *N*_1_ = {*v*_2_,*v*_4_}, *N*_3_ = {*v*_2_,*v*_4_}, $N_{1}^{+}=\left\{v_{2},v_{4}\right\}$, $N_{3}^{+}=\emptyset$, $N_{1}^{-}=\emptyset$, *and* $N_{3}^{-}=\left\{v_{2},v_{4}\right\}$. Although *v*_1_ and *v*_3_ share identical neighbor nodes, their *N* ^*+*^ and *N* ^*−*^ are different.

Therefore, we can define two distinct subsets for the DDI network: consistent neighbor nodes *S*_*c*_ and inconsistent neighbor nodes *S*_*i*_.

(2) $$S_{c}\left(v_{i},v_{j}\right)=\left(N_{i}^{+}\cap N_{j}^{+}\right)\;\cup \;\left(N_{i}^{-}\cap N_{j}^{-}\right)$$

(3) $$S_{i}\left(v_{i},v_{j}\right)=\left(N_{i}^{+}\cap N_{j}^{-}\right)\;\cup \;\left(N_{i}^{-}\cap N_{j}^{+}\right)$$

The node (drug) similarity between *v*_*i*_ and *v*_*j*_ can be defined as follows:

(4) $$\sigma_{ij}^{\text{signed}}=\frac{\left|S_{c}\left(i,j\right)\right|\;-\;|S_{i}\left(i,j\right)|}{\sqrt{{|N}_{i}|}\sqrt{{|N}_{j}|}}$$

Considering the adjacency matrix of a signed network (Equation 1), if *v*_*i*_ and *v*_*j*_ share a consistent neighbor node *v*_*k*_, then *A*_*ik*_ *A*_*kj*_ = 1. Conversely, if *v*_*i*_ and *v*_*j*_ share an inconsistent neighbor node *v*_*k*_, then *A*_*ik*_ *A*_*kj*_ = −1. If *v*_*i*_ and *v*_*j*_ do not have a common neighbor node *v*_*k*_, then *A*_*ik*_ *A*_*kj*_ = 0. Therefore, $\left|S_{c}\left(i,j\right)\right|-\left|S_{i}\left(i,j\right)\right|=\sum_{k}A_{ik}A_{kj}$. Since ${|N}_{i}|=\;\sum_{k}A_{ik}^{2}$ and ${|N}_{j}|=\;\sum_{k}A_{jk}^{2}$, we have

(5) $$\sigma_{ij}^{\text{signed}}=\frac{\sum_{k}A_{ik}A_{kj}}{\sqrt{\sum_{k}A_{ik}^{2}}\sqrt{\sum_{k}A_{jk}^{2}}}$$

**FIGURE 6:**
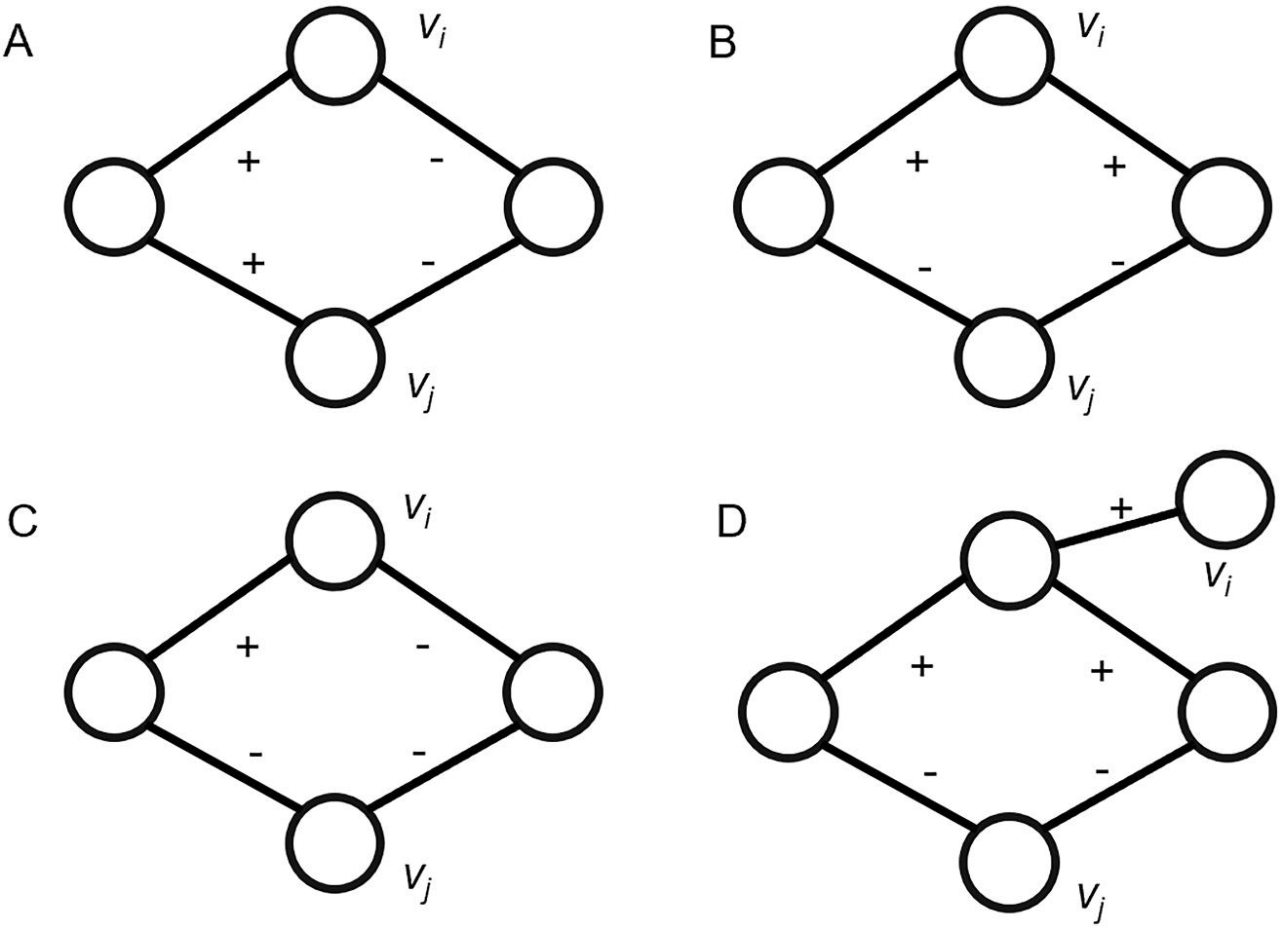
Examples of signed networks.

$\sigma_{ij}^{\text{signed}}\in \left[-1,1\right]$. $\sigma_{ij}^{\text{signed}}=1$ indicates that *v*_*i*_ and *v*_*j*_ share identical and consistent neighbor nodes (Figure 6A), and $\sigma_{ij}^{\text{signed}}=-1$ indicates that *v*_*i*_ and *v*_*j*_ share identical but inconsistent neighbor nodes (Figure 6B). When *v*_*i*_ and *v*_*j*_ share identical, half consistent, and half inconsistent neighbor nodes (Figure 6C), or *v*_*i*_ and *v*_*j*_ do not share common neighbor nodes (Figure 6D), $\sigma_{ij}^{\text{signed}}=0$. To facilitate further analysis, $\sigma_{ij}^{\text{signed}}$ can be normalized by Equation (6)

(6) $$\text{sim}\left(v_{i},v_{j}\right)=\frac{1}{1+e^{-3\sigma_{ij}^{\text{signed}}}}$$

### 4.3 Clustering analysis

For *E*. *coli* MG1655 and *E*. *coli* BW25113, we obtained a 19 × 19 similarity matrix and a 68 × 68 similarity matrix using Equation (6), respectively. Next, the similarity matrices were compressed using *t*‐SNE algorithms [34], which is a nonlinear method based on manifold learning that is widely used for dimension reduction and visualization. The parameters of *t*‐SNE were as follows: n_components = 2, perplexity = 3, learning_rate = “auto,” and n_iter = 1000. Since the relationships between drugs are tree‐like [26], a hierarchical clustering algorithm [35] was used to identify drug subgroups, thereby exploring the group–group interactions. Finally, *purity* (Equation 7) was used to evaluate the clustering quality of the DDI network.

(7) $$\begin{array}{c} purity=\frac{1}{N}\sum_{i}\sum_{j}\max\;\left(l_{ij},r_{ij}\right)\\ i=1,2,3,\text{…},k-1\\ j=i+1,i+2,\text{…},k \end{array}$$

where *l*_*ij*_ and *r*_*ij*_ are the numbers of synergistic DDI and antagonistic DDI between group *i* and group *j*, respectively. *k* is the number of clusters. *N* is the total number of DDI.

### 4.4 Prediction of unknown drug–drug interactions using semi‐supervised learning

The key to semi‐supervised learning is the affinity matrix (similarity matrix). In this study, we aimed to construct the affinity matrix using the node similarity (Equation 6). Specifically, we first divided the drug combination datasets (Tables S1,S3) into training sets (80%) and test sets (20%). The training sets were used to calculate drug similarity (Equation 6), and the test sets were used to evaluate the performance of our model. Subsequently, we employed the network projection method on the training sets to reduce the number of edge types. As a result, we obtained two subgraphs (Figure 6). For each subgraph, if two nodes are similar (*W*_*ij*_ = 1), then the two nodes have similar interactions. Therefore, we have the following loss functions:

(8) $$L\left(F^{S}\right)=\frac{1}{2}(\sum_{i,j=1}^{l+u}W_{ij}{\left\|\frac{F_{i}^{S}}{\sqrt{D_{i}}}-\frac{F_{j}^{S}}{\sqrt{D_{j}}}\right\|}^{2}+\mu \sum_{i=1}^{l}{\left\|F_{i}^{S}-Y_{i}^{S}\right\|}^{2}$$

(9) $$L\left(F^{A}\right)=\frac{1}{2}(\sum_{i,j=1}^{l+u}W_{ij}{\left\|\frac{F_{i}^{A}}{\sqrt{D_{i}}}-\frac{F_{j}^{A}}{\sqrt{D_{j}}}\right\|}^{2}+\mu \sum_{i=1}^{l}{\left\|F_{i}^{A}-Y_{i}^{A}\right\|}^{2}$$

where **F** ^*S*^ and **Y** ^*S*^ are the entire and the known synergistic drug combination matrices, respectively. **F** ^*A*^ and **Y** ^*A*^ are the entire and the known antagonistic drug combination matrices, respectively. *μ* (*μ* > 0) is a regularization parameter. **D** is a diagonal matrix and $D_{ii}=\sum_{j=1}^{l+u}W_{ij}$. **W** is the affinity matrix and is defined as follows:

(10) $$W_{ij}=\left\{\begin{array}{l} 1,\;\sigma_{ij}^{\text{signed}}\geq 0.3\;\\ 0,\;\text{otherwise}\; \end{array}\right.$$

The next step is to simplify Equations (8) and (9), respectively.

(11) $${\frac{\partial L\left(F^{S}\right)}{\partial F^{S}}|}_{F^{S}=F^{S*}}=F^{S*}-{\text{SF}}^{S*}+\mu \left(F^{S*}-Y^{S}\right)=0$$

(12) $${\frac{\partial L\left(F^{A}\right)}{\partial F^{A}}|}_{F^{A}=F^{A*}}=F^{A*}-{\text{SF}}^{A*}+\mu \left(F^{A*}-Y^{A}\right)=0$$

where **S** = **D** ^−1/2^ **WD** ^−1/2^. Since $\left(I-\frac{1}{1+\mu }S\right)$ is an invertible and positive‐definite matrix, we have the following:

(13) $$F^{S*}=\frac{\mu }{1+\mu }{\left(I-\frac{1}{1+\mu }S\right)}^{-1}Y^{S}$$

(14) $$F^{A*}=\frac{\mu }{1+\mu }{\left(I-\frac{1}{1+\mu }S\right)}^{-1}Y^{A}$$

Since Equations (13) and (14) involve matrix inversion, the time complexity is usually high. To reduce the computational burden, an iterative method can be performed.

(15) $$F^{S}\left(t+1\right)=\alpha SF^{S}\left(t\right)+\left(1-\alpha \right)Y^{S}$$

(16) $$F^{A}\left(t+1\right)=\alpha SF^{A}\left(t\right)+\left(1-\alpha \right)Y^{A}$$

where *α* (0 < *α* < 1) is a parameter and is set to 0.7 in this study. After about 30 iterations, **F** ^*A*^ and **F** ^*S*^ will converge.

Finally, we compared $F^{A*}$ and $F^{S*}$ to determine the edge type between drugs. Specifically, if $F_{ij}^{s*}>F_{ij}^{A*}$, then drug *i* and drug *j* are synergetic. Otherwise, the two drugs are antagonistic. *Precision* (Equation 17), *recall* (Equation 18), *accuracy* (Equation 19), and *F*1‐value (Equation 20) were used to evaluate the performance of our model.

(17) $$Precision=\frac{TP}{TP+FP}$$

(18) $$Recall=\frac{TP}{TP+FN}$$

(19) $$Accuracy=\frac{TP+TN}{TP+TN+FP+FN}$$

(20) $$F1=\frac{2\times Precision\times Recall}{Precision+Recall}$$

where *TP, FP, PN*, and *FN* are true positive, false positive, false negative, and true negative, respectively.

## AUTHOR CONTRIBUTIONS

**Ji Lv**: Data curation, formal analysis, methodology, visualization, writing – original draft. **Guixia Liu**: Conceptualization, funding, supervision, writing ‐ review and editing. **Yuan Ju**: writing – original draft, investigation, validation. **Houhou Huang**: Data curation, visualization, investigation. **Ying Sun**: Investigation, validation, writing – review and editing.

## CONFLICT OF INTEREST STATEMENT

The authors Ji Lv, Guixia Liu, Yuan Ju, Houhou Huang, and Ying Sun declare that they have no conflict of interest or financial conflicts to disclose.

## ETHICS STATEMENT

This article does not contain any studies with human or animal subjects performed by any of the authors.

## Supporting information

Supporting Information S1
