# Supplementary material for: Measuring drug similarity using drug–drug interactions
Source: Quant Biol. 2024 Mar 31;12(2):164–72. doi: 10.1002/qub2.38 (PMC12806202; doi:10.1002/qub2.38)
Supplement: Supplementary file 1 — Supporting Information S1 [file QUB2-12-164-s001.pdf]

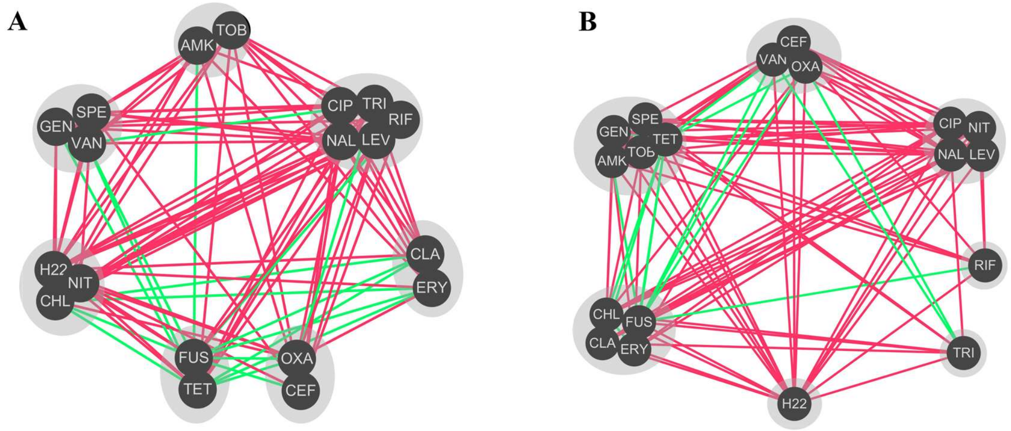

Figure S1. Clustered drug-drug interaction networks of *E. coli* MG1655 using (A) structural similarity and (B) MoA similarity. Synergistic and antagonistic drug combinations are colored in green and red, respectively.

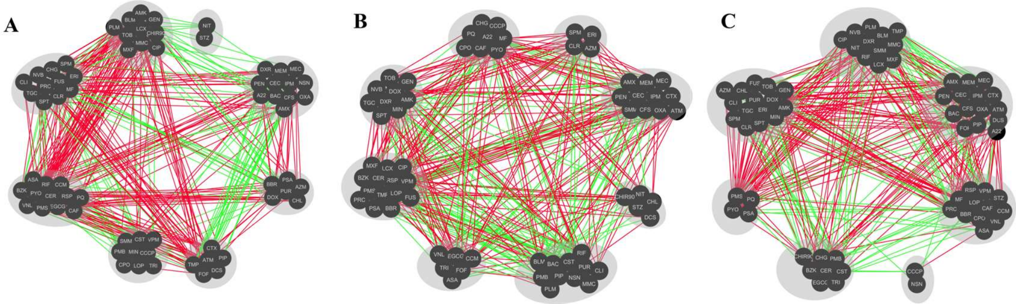

Figure S2. Clustered drug-drug interaction networks of *E. coli* BW25113 using (A) node similarity, (B) structural similarity and (C) MoA similarity. Synergistic and antagonistic drug combinations are colored in green and red, respectively.

Table S1. The test set of *E. coli* MG1655 and their predicted type using node similarity.

Drug1	Drug2	Type	FS*	FA*	Predicted type
OXA	TET	1	0.12	0.22	-1
CLA	TET	1	0.28	0.07	1
CLA	FUS	1	0.31	0.05	1
CHL	FUS	1	0.34	0.08	1
SPE	TOB	-1	0	0.36	-1
NAL	TRI	-1	0.07	0.1	-1
LEV	TOB	-1	0	0.23	-1
AMK	RIF	-1	0.01	0.2	-1
CIP	CLA	-1	0.02	0.24	-1
AMK	CHL	-1	0.05	0.3	-1
LEV	SPE	-1	0	0.24	-1
H22	TET	-1	0.15	0.3	-1
NAL	OXA	-1	0.07	0.21	-1
SPE	TRI	-1	0.1	0.19	-1
ERY	H22	-1	0	0.46	-1

CHL	GEN	-1	0.07	0.18	-1
LEV	NIT	-1	0	0.2	-1
NIT	RIF	-1	0	0.22	-1
CIP	NIT	-1	0	0.28	-1
CEF	TOB	-1	0	0.38	-1
CIP	SPE	-1	0	0.28	-1

Note: 1 and -1 represent synergistic and antagonistic drug combination, respectively.

Table S2. The test set of *E. coli* BW25113 and their predicted type using node similarity.

Drug1	Drug2	Type	FS*	FA*	Predicted type
BLM	MXF	1	0.06	0.01	1
BAC	A22	1	0.04	0.01	1
LOP	MIN	1	0.01	0	1
AMX	CAF	-1	0	0.08	-1
AMX	TMP	1	0.24	0.02	1
PYO	MMC	-1	0.02	0.16	-1
CEC	CFS	1	0.13	0	1
BZK	SMM	1	0.05	0	1
AMX	CEC	1	0.08	0.01	1
BAC	CST	1	0.03	0.02	1
PRC	PLM	1	0	0.02	-1
PYO	MXF	-1	0	0.06	-1
BAC	CFS	1	0.05	0	1
PRC	BLM	1	0	0.15	-1
CHL	CER	-1	0.01	0.04	-1
PYO	GEN	-1	0.01	0.07	-1
MEC	NSN	1	0	0	-1
EGCG	PLM	-1	0	0.1	-1
DOX	MEC	-1	0.07	0.03	1
CEC	A22	1	0.13	0.02	1
A22	MF	-1	0	0.12	-1
MEC	DXR	1	0.02	0	1
CEC	PUR	-1	0.01	0.04	-1
CLI	PYO	-1	0.01	0.12	-1
RIF	MF	-1	0	0.09	-1
PMB	CEC	-1	0.01	0.01	-1
PMS	PUR	-1	0.03	0.05	-1
PMB	CLI	1	0	0.01	-1
OXA	PMB	-1	0.02	0.02	-1
BZK	TMP	-1	0.05	0.11	-1
CER	CST	1	0.09	0	1
LOP	CPO	1	0	0	-1
TMP	CLR	-1	0.03	0.06	-1
CIP	CAF	-1	0	0.01	-1

CEC	PRC	-1	0.01	0.11	-1
TMP	PYO	-1	0.01	0.23	-1
A22	MMC	1	0.05	0.12	-1
RIF	CCM	1	0	0.02	-1
SPM	MF	-1	0.02	0.09	-1
AMX	PYO	-1	0.02	0.11	-1
BAC	TMP	1	0.12	0.02	1
BAC	MEM	1	0.11	0	1
PRC	CLR	1	0	0.09	-1
FOF	NVB	-1	0.01	0.09	-1
PRC	ATM	-1	0	0.05	-1
PSA	IPM	-1	0.06	0.06	-1
DOX	CER	-1	0	0.01	-1
CER	CHIR90	-1	0.03	0.03	-1
PRC	MMC	1	0.01	0.02	-1
BAC	BZK	-1	0.01	0.12	-1
AMK	MMC	1	0.05	0.12	-1
CIP	FOF	-1	0.01	0.18	-1
ATM	PYO	-1	0.01	0.31	-1
BLM	ASA	-1	0.04	0.07	-1
CIP	PMS	-1	0.01	0.23	-1
PMS	CHIR90	-1	0.03	0.14	-1
CLI	TOB	-1	0.01	0.11	-1
CTX	CFS	1	0.17	0	1
AMK	CLI	-1	0.01	0.06	-1
PMS	PLM	-1	0	0.12	-1
CCCP	LOP	1	0.15	0	1
LOP	CST	1	0.14	0.01	1
PYO	ASA	1	0.13	0.02	1
DOX	FOF	-1	0.01	0.17	-1
FUS	BZK	-1	0.01	0.1	-1
DOX	IPM	-1	0.02	0.01	1
BAC	MMC	1	0.02	0.04	-1
EGCG	CPO	1	0	0	-1
CTX	PRC	-1	0.06	0.12	-1
LCX	RIF	-1	0.07	0.14	-1
DOX	A22	-1	0.02	0.03	-1
TMP	CHIR90	-1	0.03	0.01	1
CIP	BZK	-1	0.02	0.08	-1
CFS	PIP	1	0.02	0.01	1
CIP	VNL	-1	0.02	0.16	-1
VPM	CST	1	0.06	0.01	1
OXA	PEN	1	0.06	0	1

Note: 1 and -1 represent synergistic and antagonistic drug combination, respectively.

Table S3. The test set of *E. coli* MG1655 and their predicted type using structural similarity.

Drug1	Drug2	Type	FS*	FA*	Predicted type
OXA	TET	1	0	0	-1
CLA	TET	1	0.32	0.04	1
CLA	FUS	1	0.37	0	1
CHL	FUS	1	0	0	-1
SPE	TOB	-1	0	0.25	-1
NAL	TRI	-1	0	0	-1
LEV	TOB	-1	0	0.51	-1
AMK	RIF	-1	0	0.22	-1
CIP	CLA	-1	0	0.26	-1
AMK	CHL	-1	0.11	0.12	-1
LEV	SPE	-1	0	0	-1
H22	TET	-1	0	0	-1
NAL	OXA	-1	0	0	-1
SPE	TRI	-1	0.1	0.15	-1
ERY	H22	-1	0	0.5	-1
CHL	GEN	-1	0	0	-1
LEV	NIT	-1	0	0	-1
NIT	RIF	-1	0	0	-1
CIP	NIT	-1	0	0	-1
CEF	TOB	-1	0	0	-1
CIP	SPE	-1	0	0	-1

Note: 1 and -1 represent synergistic and antagonistic drug combination, respectively.

Table S4. The test set of *E. coli* BW25113 and their predicted type using structural similarity.

Drug1	Drug2	Type	FS*	FA*	Predicted type
BLM	MXF	1	0.06	0.01	1
BAC	A22	1	0.04	0.01	1
LOP	MIN	1	0.01	0	1
AMX	CAF	-1	0	0.08	-1
AMX	TMP	1	0.24	0.02	1
PYO	MMC	-1	0.02	0.16	-1
CEC	CFS	1	0.13	0	1
BZK	SMM	1	0.05	0	1
AMX	CEC	1	0.08	0.01	1
BAC	CST	1	0.03	0.02	1
PRC	PLM	1	0	0.02	-1
PYO	MXF	-1	0	0.06	-1
BAC	CFS	1	0.05	0	1
PRC	BLM	1	0	0.15	-1
CHL	CER	-1	0.01	0.04	-1
PYO	GEN	-1	0.01	0.07	-1
MEC	NSN	1	0	0	-1
EGCG	PLM	-1	0	0.1	-1

DOX	MEC	-1	0.07	0.03	1
CEC	A22	1	0.13	0.02	1
A22	MF	-1	0	0.12	-1
MEC	DXR	1	0.02	0	1
CEC	PUR	-1	0.01	0.04	-1
CLI	PYO	-1	0.01	0.12	-1
RIF	MF	-1	0	0.09	-1
PMB	CEC	-1	0.01	0.01	-1
PMS	PUR	-1	0.03	0.05	-1
PMB	CLI	1	0	0.01	-1
OXA	PMB	-1	0.02	0.02	-1
BZK	TMP	-1	0.05	0.11	-1
CER	CST	1	0.09	0	1
LOP	CPO	1	0	0	-1
TMP	CLR	-1	0.03	0.06	-1
CIP	CAF	-1	0	0.01	-1
CEC	PRC	-1	0.01	0.11	-1
TMP	PYO	-1	0.01	0.23	-1
A22	MMC	1	0.05	0.12	-1
RIF	CCM	1	0	0.02	-1
SPM	MF	-1	0.02	0.09	-1
AMX	PYO	-1	0.02	0.11	-1
BAC	TMP	1	0.12	0.02	1
BAC	MEM	1	0.11	0	1
PRC	CLR	1	0	0.09	-1
FOF	NVB	-1	0.01	0.09	-1
PRC	ATM	-1	0	0.05	-1
PSA	IPM	-1	0.06	0.06	-1
DOX	CER	-1	0	0.01	-1
CER	CHIR90	-1	0.03	0.03	-1
PRC	MMC	1	0.01	0.02	-1
BAC	BZK	-1	0.01	0.12	-1
AMK	MMC	1	0.05	0.12	-1
CIP	FOF	-1	0.01	0.18	-1
ATM	PYO	-1	0.01	0.31	-1
BLM	ASA	-1	0.04	0.07	-1
CIP	PMS	-1	0.01	0.23	-1
PMS	CHIR90	-1	0.03	0.14	-1
CLI	TOB	-1	0.01	0.11	-1
CTX	CFS	1	0.17	0	1
AMK	CLI	-1	0.01	0.06	-1
PMS	PLM	-1	0	0.12	-1
CCCP	LOP	1	0.15	0	1
LOP	CST	1	0.14	0.01	1

PYO	ASA	1	0.13	0.02	1
DOX	FOF	-1	0.01	0.17	-1
FUS	BZK	-1	0.01	0.1	-1
DOX	IPM	-1	0.02	0.01	1
BAC	MMC	1	0.02	0.04	-1
EGCG	CPO	1	0	0	-1
CTX	PRC	-1	0.06	0.12	-1
LCX	RIF	-1	0.07	0.14	-1
DOX	A22	-1	0.02	0.03	-1
TMP	CHIR90	-1	0.03	0.01	1
CIP	BZK	-1	0.02	0.08	-1
CFS	PIP	1	0.02	0.01	1
CIP	VNL	-1	0.02	0.16	-1
VPM	CST	1	0.06	0.01	1
OXA	PEN	1	0.06	0	1

Note: 1 and -1 represent synergistic and antagonistic drug combination, respectively.

Table S5. The test set of *E. coli* MG1655 and their predicted type using MoA similarity.

Drug1	Drug2	Type	FS*	FA*	Predicted type
OXA	TET	1	0	0.175	-1
CLA	TET	1	0.175	0	1
CLA	FUS	1	0	0	-1
CHL	FUS	1	0.175	0	1
SPE	TOB	-1	0	0	-1
NAL	TRI	-1	0	0	-1
LEV	TOB	-1	0	0	-1
AMK	RIF	-1	0	0	-1
CIP	CLA	-1	0	0.13	-1
AMK	CHL	-1	0	0	-1
LEV	SPE	-1	0	0	-1
H22	TET	-1	0	0.175	-1
NAL	OXA	-1	0	0	-1
SPE	TRI	-1	0	0	-1
ERY	H22	-1	0	0.175	-1
CHL	GEN	-1	0.175	0	1
LEV	NIT	-1	0	0	-1
NIT	RIF	-1	0	0.055	-1
CIP	NIT	-1	0	0.13	-1
CEF	TOB	-1	0	0	-1
CIP	SPE	-1	0	0.13	-1

Note: 1 and -1 represent synergistic and antagonistic drug combination, respectively.

Table S6. The test set of *E. coli* BW25113 and their predicted type using MoA similarity.

Drug1	Drug2	Type	FS*	FA*	Predicted type
BLM	MXF	1	0.06	0	1
BAC	A22	1	0.19	0	1
LOP	MIN	1	0	0	-1
AMX	CAF	-1	0	0.14	-1
AMX	TMP	1	0.23	0.09	1
PYO	MMC	-1	0	0.17	-1
CEC	CFS	1	0.28	0	1
BZK	SMM	1	0	0	-1
AMX	CEC	1	0.19	0	1
BAC	CST	1	0	0.05	-1
PRC	PLM	1	0	0	-1
PYO	MXF	-1	0	0.17	-1
BAC	CFS	1	0.28	0	1
PRC	BLM	1	0.06	0.17	-1
CHL	CER	-1	0	0.05	-1
PYO	GEN	-1	0	0.17	-1
MEC	NSN	1	0	0	-1
EGCG	PLM	-1	0	0	-1
DOX	MEC	-1	0	0.23	-1
CEC	A22	1	0.19	0	1
A22	MF	-1	0	0.14	-1
MEC	DXR	1	0.05	0	1
CEC	PUR	-1	0	0.14	-1
CLI	PYO	-1	0	0.37	-1
RIF	MF	-1	0	0.06	-1
PMB	CEC	-1	0	0	-1
PMS	PUR	-1	0	0.17	-1
PMB	CLI	1	0	0	-1
OXA	PMB	-1	0	0	-1
BZK	TMP	-1	0	0	-1
CER	CST	1	0.09	0	1
LOP	CPO	1	0	0	-1
TMP	CLR	-1	0	0.12	-1
CIP	CAF	-1	0	0	-1
CEC	PRC	-1	0	0.19	-1
TMP	PYO	-1	0	0.23	-1
A22	MMC	1	0.05	0.19	-1
RIF	CCM	1	0	0.06	-1
SPM	MF	-1	0	0.28	-1
AMX	PYO	-1	0.05	0.14	-1
BAC	TMP	1	0.23	0.09	1
BAC	MEM	1	0.14	0	1

PRC	CLR	1	0	0.06	-1
FOF	NVB	-1	0.05	0.14	-1
PRC	ATM	-1	0.06	0.29	-1
PSA	IPM	-1	0	0	-1
DOX	CER	-1	0	0.05	-1
CER	CHIR90	-1	0	0	-1
PRC	MMC	1	0.06	0	1
BAC	BZK	-1	0	0.28	-1
AMK	MMC	1	0	0.05	-1
CIP	FOF	-1	0	0.23	-1
ATM	PYO	-1	0.05	0.14	-1
BLM	ASA	-1	0.06	0.06	-1
CIP	PMS	-1	0.06	0.35	-1
PMS	CHIR90	-1	0	0.35	-1
CLI	TOB	-1	0.09	0.14	-1
CTX	CFS	1	0.28	0	1
AMK	CLI	-1	0	0.05	-1
PMS	PLM	-1	0	0.17	-1
CCCP	LOP	1	0	0	-1
LOP	CST	1	0	0	-1
PYO	ASA	1	0.17	0	1
DOX	FOF	-1	0	0.28	-1
FUS	BZK	-1	0	0.09	-1
DOX	IPM	-1	0	0.14	-1
BAC	MMC	1	0.05	0.19	-1
EGCG	CPO	1	0	0	-1
CTX	PRC	-1	0	0.19	-1
LCX	RIF	-1	0.06	0.29	-1
DOX	A22	-1	0	0.09	-1
TMP	CHIR90	-1	0	0	-1
CIP	BZK	-1	0	0.06	-1
CFS	PIP	1	0.05	0	1
CIP	VNL	-1	0	0.12	-1
VPM	CST	1	0	0	-1
OXA	PEN	1	0.09	0	1

Note: 1 and -1 represent synergistic and antagonistic drug combination, respectively.

Table S7. Drug combinations listed in *E. coli* MG1655 and their type.

Drug1	Drug2	Type	Drug1	Drug2	Type
FUS	VAN	1	ERY	LEV	0
CLA	FUS	1	OXA	RIF	0
OXA	TRI	1	CLA	TOB	0
ERY	FUS	1	CIP	FUS	0
FUS	OXA	1	AMK	VAN	-1

TRI	VAN	1	CEF	CIP	-1
TET	VAN	1	FUS	LEV	-1
CLA	TET	1	AMK	RIF	-1
FUS	RIF	1	CLA	NAL	-1
CHL	FUS	1	CEF	RIF	-1
OXA	TET	1	ERY	OXA	-1
ERY	TET	1	CIP	GEN	-1
LEV	NAL	1	NAL	VAN	-1
CEF	FUS	1	FUS	NIT	-1
CHL	ERY	1	LEV	NIT	-1
CHL	CLA	1	CHL	OXA	-1
CHL	TET	1	NAL	TRI	-1
GEN	TET	1	TET	TOB	-1
AMK	FUS	1	CHL	NIT	-1
FUS	TET	1	LEV	OXA	-1
CIP	TRI	0	AMK	CEF	-1
AMK	NIT	0	CLA	LEV	-1
AMK	TRI	0	CIP	ERY	-1
CEF	OXA	0	AMK	ERY	-1
CLA	RIF	0	GEN	NAL	-1
AMK	CLA	0	NAL	OXA	-1
RIF	TET	0	GEN	RIF	-1
NIT	TRI	0	ERY	TOB	-1
OXA	VAN	0	CEF	TOB	-1
CLA	ERY	0	TOB	VAN	-1
GEN	TOB	0	NIT	TET	-1
CHL	SPE	0	FUS	TRI	-1
LEV	TRI	0	SPE	TRI	-1
CLA	VAN	0	LEV	RIF	-1
CEF	TET	0	CIP	NIT	-1
AMK	CIP	0	NIT	SPE	-1
CIP	LEV	0	ERY	NAL	-1
GEN	NIT	0	CHL	GEN	-1
ERY	VAN	0	NAL	NIT	-1
GEN	OXA	0	H22	TOB	-1
CHL	VAN	0	CEF	NAL	-1
GEN	LEV	0	CIP	CLA	-1
CEF	ERY	0	AMK	H22	-1
ERY	RIF	0	TET	TRI	-1
OXA	SPE	0	AMK	SPE	-1
NIT	TOB	0	CHL	CIP	-1
CEF	CLA	0	FUS	H22	-1
CEF	TRI	0	CEF	SPE	-1
CLA	NIT	0	LEV	TOB	-1

SPE	TET	0	H22	TET	-1
ERY	SPE	0	GEN	SPE	-1
CIP	NAL	0	CHL	TRI	-1
CHL	RIF	0	CHL	NAL	-1
CLA	SPE	0	LEV	TET	-1
CEF	GEN	0	AMK	CHL	-1
LEV	VAN	0	H22	OXA	-1
RIF	SPE	0	NAL	SPE	-1
TOB	TRI	0	H22	SPE	-1
NIT	VAN	0	NAL	TOB	-1
RIF	TRI	0	CEF	NIT	-1
ERY	GEN	0	CLA	TRI	-1
FUS	SPE	0	CIP	TOB	-1
NAL	RIF	0	GEN	H22	-1
CLA	GEN	0	NIT	RIF	-1
FUS	TOB	0	CEF	LEV	-1
AMK	TOB	0	CEF	H22	-1
AMK	OXA	0	LEV	SPE	-1
CIP	VAN	0	NAL	TET	-1
ERY	NIT	0	RIF	TOB	-1
OXA	TOB	0	CHL	H22	-1
GEN	TRI	0	NIT	OXA	-1
AMK	LEV	0	ERY	H22	-1
CIP	RIF	0	CHL	TOB	-1
FUS	GEN	0	H22	VAN	-1
AMK	NAL	0	CIP	SPE	-1
FUS	NAL	0	H22	NAL	-1
AMK	GEN	0	H22	LEV	-1
CLA	OXA	0	CLA	H22	-1
CEF	CHL	0	H22	NIT	-1
CIP	OXA	0	CIP	H22	-1
AMK	TET	0	CIP	TET	-1
RIF	VAN	0	H22	RIF	-1
CEF	VAN	0	SPE	TOB	-1
SPE	VAN	0	CHL	LEV	-1
ERY	TRI	0	H22	TRI	-1
GEN	VAN	0			

Note: 1, 0 and -1 represent synergistic, additive and antagonistic drug combination, respectively.

Table S8. Drugs listed in *E. coli* BW25113 and their category, targeted cellular process, class and abbreviation.

Drug	Drug category	Targeted cellular process	Class	Abbreviation
Amikacin	aminoglycoside	protein synthesis inhibitor	antibiotic	AMK
Amoxicillin	beta-lactam	cell wall	antibiotic	AMX
Aztreonam	beta-lactam	cell wall	antibiotic	ATM
Azithromycin	macrolide	protein synthesis inhibitor	antibiotic	AZM
Bacitracin	other cell wall	cell wall	antibiotic	BAC
Cefaclor	beta-lactam	cell wall	antibiotic	CEC
Cefsulodin	beta-lactam	cell wall	antibiotic	CFS
Chloramphenicol	protein synthesis inhibitor	protein synthesis inhibitor	antibiotic	CHL
Ciprofloxacin	quinolones	DNA	antibiotic	CIP
Clindamycin	protein synthesis inhibitor	protein synthesis inhibitor	antibiotic	CLI
Clarithromycin	macrolide	protein synthesis inhibitor	antibiotic	CLR
Colistin	LPS	membrane	antibiotic	CST
Cefotaxime	beta-lactam	cell wall	antibiotic	CTX
Cycloserine D	other cell wall	cell wall	antibiotic	DCS
Doxycycline	tetracycline	protein synthesis inhibitor	antibiotic	DOX
Erythromycin	macrolide	protein synthesis inhibitor	antibiotic	ERI
Fosfomycin	other cell wall	cell wall	antibiotic	FOF
Fusidic acid	protein synthesis inhibitor	protein synthesis inhibitor	antibiotic	FUS
Gentamicin	aminoglycoside	protein synthesis inhibitor	antibiotic	GEN
Imipenem	beta-lactam	cell wall	antibiotic	IPM
Levofloxacin	DNA gyrase	DNA	antibiotic	LCX
Mecillinam	beta-lactam	cell wall	antibiotic	MEC
Meropenem	beta-lactam	cell wall	antibiotic	MEM
Minocycline	tetracycline	protein synthesis inhibitor	antibiotic	MIN
Moxifloxacin	quinolones	DNA	antibiotic	MXF
Nitrofurantoin	multiple	DNA	antibiotic	NIT
Nisin	PMF	PMF	antibiotic	NSN
Novobiocin	quinolones	DNA	antibiotic	NVB
Oxacillin	beta-lactam	cell wall	antibiotic	OXA
Penicillin G	beta-lactam	cell wall	antibiotic	PEN
Piperacillin	beta-lactam	cell wall	antibiotic	PIP
Polymyxin B	LPS	membrane	antibiotic	PMB
Puromycin	protein synthesis inhibitor	protein synthesis inhibitor	antibiotic	PUR
Rifampicin	RNA polymerase	DNA	antibiotic	RIF
Sulfamonomethoxine	folic acid biosynthesis	DNA	antibiotic	SMM
Spiramycin	macrolide	protein synthesis inhibitor	antibiotic	SPM
Spectinomycin	aminoglycoside	protein synthesis inhibitor	antibiotic	SPT
Tigecycline	tetracycline	protein synthesis inhibitor	antibiotic	TGC
Trimethoprim	folic acid biosynthesis	DNA	antibiotic	TMP
Tobramycin	aminoglycoside	protein synthesis inhibitor	antibiotic	TOB
Cerulenin	fatty acid biosynthesis	membrane	anti-infective	CER

Triclosan	other membrane	membrane	anti-infective	TRI
Benzalkonium	other membrane	membrane	antiseptic	BZK
Chlorhexidine	LPS	membrane	antiseptic	CHG
Berberine	food additive	human	food additive	BBR
Caffeine	food additive	human	food additive	CAF
Curcumin	food additive	human	food additive	CCM
EGCG	fatty acid biosynthesis	membrane	food additive	EGCG
Vanillin	food additive	human	food additive	VNL
Acetylsalisylic acid	human drug	human	human-targeted drug	ASA
Bleomycin	multiple	DNA	human-targeted drug	BLM
Ciclopirox	human drug	human	human-targeted drug	CPO
Doxorubicin	other DNA	DNA	human-targeted drug	DXR
Loperamide	human drug	human	human-targeted drug	LOP
Metformin	human drug	human	human-targeted drug	MF
Phleomycin	multiple	DNA	human-targeted drug	PLM
Procaine	human drug	human	human-targeted drug	PRC
Reserpine	human drug	human	human-targeted drug	RSP
Streptozotocin	human drug	human	human-targeted drug	STZ
Verapamil	human drug	human	human-targeted drug	VPM
A22	other cell wall	cell wall	research used drug	A22
CCCP	PMF	PMF	research used drug	CCCP
CHIR-90	LPS	membrane	research used drug	CHIR90
Mitomycin C	other DNA	DNA	research used drug	MMC
PMS	oxidative stress	oxidative stress	research used drug	PMS
Paraquat	oxidative stress	oxidative stress	research used drug	PQ
Pseudomonic acid	oxidative stress	oxidative stress	research used drug	PSA
Pyocyanin	oxidative stress	oxidative stress	research used drug	PYO

Table S9. Drug combinations listed in *E. coli* BW25113 and their type.

Drug1	Drug2	Type	Drug1	Drug2	Type	Drug1	Drug2	Type
AMX	OXA	1	SPT	TMP	-1	CHL	MXF	0
AMX	CFS	1	SPT	MF	-1	CIP	CLR	0
AMX	TMP	1	SPT	PLM	-1	DOX	PQ	0
AMX	ASA	1	OXA	BZK	-1	DOX	ATM	0
CHL	FUS	1	NVB	CER	-1	DOX	PEN	0
CHL	RIF	1	NVB	CCM	-1	DOX	CLO	0
CIP	BAC	1	AMK	BZK	-1	DOX	STZ	0
DOX	TOB	1	CEC	PRC	-1	SPT	EGCG	0
DOX	CLR	1	CEC	VNL	-1	SPT	PUR	0
OXA	CEC	1	CLI	CLR	-1	NVB	PLM	0
OXA	MEC	1	PQ	CHIR90	-1	BAC	RSP	0
BAC	CEC	1	PRC	ATM	-1	PMB	PLM	0
BAC	CFS	1	PRC	GEN	-1	AMK	CCM	0
BAC	A22	1	LCX	ASA	-1	CLI	MMC	0

BAC	MMC	1	MEC	MF	-1	ERI	PLM	0
BAC	MEM	1	EGCG	PLM	-1	TMP	PQ	0
PMB	CLI	1	RIF	ATM	-1	TMP	VNL	0
PMB	CCM	1	ATM	CAF	-1	TMP	PIP	0
PMB	TRI	1	ATM	ASA	-1	TMP	MF	0
FUS	PUR	1	CLR	BLM	-1	PQ	LCX	0
CTX	CFS	1	CLR	PLM	-1	PQ	MF	0
BZK	SMM	1	CER	CHIR90	-1	PQ	PLM	0
CEC	CFS	1	CIP	PQ	-1	PRC	MMC	1
CEC	BLM	1	VNL	CCCP	-1	VNL	PIP	0
CFS	MEC	1	VNL	MXF	-1	VNL	MEC	0
CFS	IPM	1	EGCG	MXF	-1	PIP	RSP	0
CLI	TMP	1	DCS	PIP	0	NIT	MMC	0
SPM	NSN	1	DOX	AZM	0	MEC	MMC	1
SPM	CST	1	FOF	BAC	0	PEN	CLR	0
ERI	EGCG	1	FOF	PIP	0	PEN	MMC	0
TMP	VPM	1	NVB	CFS	0	CLR	PUR	0
TMP	MEC	1	NVB	A22	0	BLM	MMC	0
TMP	IPM	1	NVB	BLM	0	DOX	BAC	0
SMM	RIF	1	PMB	CCCP	0	PEN	PUR	0
PRC	PUR	1	FUS	CCCP	0	AMX	PMB	0
PRC	BLM	1	CTX	CEC	0	CHL	PQ	0
VPM	A22	1	SPM	CHIR90	0	CIP	ERI	0
VPM	LOP	1	ERI	CHIR90	0	DCS	TOB	0
NIT	BLM	1	PQ	IPM	0	NVB	CHIR90	0
MEC	A22	1	VNL	CPO	0	AMK	LOP	0
MEC	BLM	1	VNL	PLM	0	BZK	TOB	0
A22	BLM	1	VPM	CHG	0	CEC	CST	0
LOP	MIN	1	PIP	MEM	0	CFS	A22	0
LOP	CPO	1	PIP	CHIR90	0	PQ	CCCP	0
MIN	EGCG	1	LOP	CHG	0	NIT	MEC	0
TOB	PUR	1	MTZ	MMC	0	AMX	IPM	0
EGCG	CPO	1	RIF	CHIR90	0	CHL	CCCP	0
RIF	CCM	1	BLM	CHIR90	0	CHL	LEX	0
ATM	PEN	1	DOX	RIF	0	DCS	MEM	0
CIP	AMK	1	DOX	CHIR90	0	FOF	AMK	0
CFS	LCX	1	NVB	AMK	0	OXA	A22	0
TMP	MEM	1	AMK	LCX	0	NVB	MEC	0
VPM	ATM	1	CLI	CHIR90	0	NVB	TOB	0
MEC	NSN	1	CHL	TMP	0	NVB	MF	0
MEC	DXR	1	CHL	MMC	0	PMB	CER	0
ATM	DXR	1	DOX	PLM	0	FUS	TOB	0
PUR	GEN	1	SPT	NVB	0	AMK	CEC	0
BLM	MXF	1	NVB	PQ	0	CEC	TOB	0

AMX	DOX	-1	NVB	PRC	0	TMP	EGCG	0
AMX	PRC	-1	NVB	VNL	0	TMP	STZ	0
AMX	VNL	-1	NVB	NIT	0	PRC	CER	0
AMX	CAF	-1	NVB	CLR	0	CCCP	CHG	0
AMX	MF	-1	NVB	RSP	0	CCCP	RIF	0
CHL	MEC	-1	FUS	MMC	0	CCCP	CLR	0
CHL	IPM	-1	BZK	GEN	0	A22	PEN	0
CHL	MF	-1	TMP	PRC	0	LOP	CHIR90	0
CIP	PRC	-1	TMP	ATM	0	MIN	CHIR90	0
DCS	PLM	-1	TMP	PEN	0	CHG	CLR	0
DOX	CTX	-1	TMP	PUR	0	EGCG	RSP	0
SPT	FOF	-1	TMP	AZM	0	RIF	CAF	0
SPT	PRC	-1	TMP	CCM	0	RIF	ASA	1
SPT	BLM	-1	PQ	MMC	0	BLM	PHM	0
FOF	BZK	-1	PQ	AZM	0	DCS	CEC	0
FOF	TMP	-1	PQ	MXF	0	DCS	CCCP	0
FOF	TOB	-1	MEC	RIF	0	NVB	IPM	0
FOF	RIF	-1	MEC	CST	0	CEC	RSP	0
FOF	MMC	-1	ATM	GEN	0	CEC	MEM	0
FOF	CPO	-1	CLR	MMC	0	LCX	RSP	0
FOF	CHIR90	-1	PUR	MMC	0	CCCP	MEC	0
NVB	CEC	-1	CHL	CPO	0	MEC	CAF	0
NVB	RIF	-1	PQ	PUR	0	MEC	IPM	0
NVB	ATM	-1	AMX	AMK	1	MEC	MEM	0
FUS	BZK	-1	FOF	PQ	1	PEN	MEM	0
FUS	TMP	-1	NVB	MMC	1	AMX	CPO	0
FUS	MEC	-1	NVB	CST	1	CHL	NVB	0
FUS	CCM	-1	BAC	CST	1	CHL	CLO	0
CTX	TMP	-1	PMB	BZK	1	CIP	FUS	0
CTX	PRC	-1	PMB	VPM	1	CIP	PEN	0
CTX	CAF	-1	PMB	LOP	1	CIP	CST	0
CTX	MMC	-1	PMB	CLR	1	DCS	DOX	0
CTX	CCM	-1	FUS	CST	1	DOX	LCX	0
BZK	TMP	-1	BZK	CCCP	1	DOX	CCM	0
BZK	MEC	-1	BZK	CHG	1	SPT	CLI	0
CEC	SPM	-1	BZK	CST	1	SPT	CPO	0
CEC	ERI	-1	ERI	CST	1	SPT	MXF	0
CEC	CAF	-1	VPM	MMC	1	NVB	ERI	0
CEC	PUR	-1	VPM	CST	1	NVB	DIC	0
CLI	BLM	-1	A22	IPM	1	NVB	CPO	0
SPM	MF	-1	LOP	CST	1	FUS	PQ	0
ERI	IPM	-1	RIF	CST	1	CEC	MF	0
ERI	BLM	-1	CLR	CST	1	CLI	ERI	0
ERI	MF	-1	DOX	CHG	1	ERI	PQ	0

ERI	ASA	-1	CHL	A22	-1	ERI	PUR	0
ERI	GEN	-1	CIP	BZK	-1	ERI	MXF	0
TMP	CLR	-1	CIP	MIN	-1	TMP	CST	0
TMP	CHIR90	-1	AMK	ERI	-1	PIP	MXF	0
PRC	TOB	-1	AMK	PRC	-1	MEC	CHG	0
PRC	IPM	-1	AMK	EGCG	-1	MEC	CPO	0
VNL	BLM	-1	AMK	CLR	-1	A22	MXF	0
VNL	CHIR90	-1	AMK	MF	-1	A22	GEN	0
LCX	CST	-1	AMK	BBR	-1	CLO	MXF	0
MEC	CLR	-1	VNL	ATM	-1	CLR	MXF	0
MEC	PUR	-1	ATM	MMC	-1	PUR	AZM	0
MEC	AZM	-1	MMC	AZM	-1	IPM	MXF	0
MEC	CCM	-1	PIP	MMC	-1	AMX	BZK	0
A22	PUR	-1	CIP	SPT	-1	CIP	PMB	0
A22	MF	-1	FUS	PRC	0	DCS	MXF	0
A22	CCM	-1	AMK	PIP	0	DCS	GEN	0
A22	ASA	-1	CTX	A22	0	FOF	EGCG	0
LOP	BLM	-1	PIP	A22	0	PMB	MEC	0
TOB	MF	-1	DXR	MMC	0	CEC	CPO	0
EGCG	ATM	-1	CIP	LCX	0	ERI	TEC	0
RIF	BLM	-1	PMB	RIF	0	SMM	A22	0
RIF	MF	-1	CIP	DOX	0	PQ	MEC	0
RIF	GEN	-1	DOX	MMC	0	PQ	A22	0
ATM	BLM	-1	SPT	CLR	0	VNL	A22	0
ATM	AZM	-1	AMX	TOB	0	LCX	CPO	0
ATM	CCM	-1	CHL	PRC	0	PIP	CLO	0
ATM	BBR	-1	DOX	NIT	0	NIT	ATM	0
CLR	CER	-1	FOF	TGC	0	CHG	ATM	0
CLR	MF	-1	OXA	TOB	0	CLO	CHIR90	0
BLM	MF	-1	BAC	TOB	0	DOX	PRC	0
BLM	ASA	-1	BAC	CHG	0	DOX	MF	0
DCS	PRC	-1	PMB	PRC	0	FOF	VPM	0
AMX	SPT	-1	PMB	CHG	0	NVB	CHG	0
CIP	CAF	-1	CTX	CLR	0	AMK	ATM	0
DCS	ERI	-1	CTX	MF	0	BZK	VPM	0
DCS	CHG	-1	ERI	ATM	0	BZK	MMC	0
OXA	PMB	-1	SMM	MIN	0	MIN	CHG	0
PMB	CEC	-1	PQ	LOP	0	CHG	MMC	0
AMK	CLI	-1	PRC	LCX	0	CAF	MMC	0
AMK	SPM	-1	PRC	MIN	0	FOF	ATM	0
CTX	RSP	-1	LCX	NIT	0	AMK	TOB	0
CLI	TOB	-1	NIT	RIF	0	CEC	PEN	0
PQ	BLM	-1	A22	CHG	0	LCX	CHG	0
TOB	CHG	-1	LOP	TEC	0	LCX	MF	0

TOB	TGC	-1	LOP	CLO	0	PIP	TOB	0
PUR	IPM	-1	LOP	CLR	0	PIP	ATM	0
AMX	PEN	0	MIN	RSP	0	CHL	CIP	0
CHL	STZ	0	CHG	CAF	0	CIP	EGCG	0
DOX	FUS	0	EGCG	CLR	0	SPT	CHG	0
DOX	ERI	0	EGCG	MMC	0	AMK	CHG	0
FOF	LOP	0	PEN	MXF	0	PQ	ATM	0
OXA	CTX	0	IPM	PHM	0	LCX	MMC	0
OXA	PIP	0	IPM	TRI	0	IPM	MMC	0
OXA	ATM	0	AMX	GEN	0	MMC	MEM	0
OXA	CHIR90	0	CIP	CTX	0	MMC	GEN	0
BAC	CTX	0	CIP	PIP	0	DOX	MXF	0
BAC	CAF	0	CIP	MF	0	SPT	AMK	0
PMB	TMP	0	BAC	GEN	0	PQ	PIP	0
PMB	NIT	0	AMK	IPM	0	BAC	PMB	0
FUS	CTX	0	AMK	MEM	0	CHG	CER	0
FUS	VPM	0	CTX	LCX	0	TOB	ATM	0
FUS	PIP	0	CTX	TOB	0	AMX	CIP	0
FUS	NIT	0	CTX	GEN	0	AMX	CTX	1
FUS	CLR	0	BZK	PQ	0	AMX	PIP	1
AMK	TMP	0	ERI	PIP	0	NVB	MIN	1
CTX	ATM	0	LCX	PIP	0	FUS	AMK	1
CTX	PEN	0	PIP	NIT	0	AMK	NIT	1
CEC	TMP	0	PIP	CLR	0	AMK	MMC	1
CEC	PQ	0	LOP	GEN	0	CFS	PIP	1
CFS	PEN	0	MIN	CST	0	TMP	A22	1
ERI	RIF	0	TEC	RIF	0	PRC	CLR	1
TMP	CLO	0	CHG	CST	0	VPM	RIF	1
TMP	TGC	0	CHG	MXF	0	NIT	TOB	1
SMM	CLR	0	ATM	CLR	0	NIT	IPM	1
SMM	BLM	0	CIP	LOP	0	A22	MMC	1
PQ	NIT	0	CIP	PUR	0	CIP	FOF	-1
PRC	BBR	0	CIP	AZM	0	SPT	ATM	-1
VPM	CLR	0	CIP	BBR	0	FOF	NVB	-1
PIP	PEN	0	CIP	PLM	0	FOF	ERI	-1
NIT	CLR	0	DCS	CTX	0	FOF	LCX	-1
MIN	CCM	0	SPT	NIT	0	FOF	CLR	-1
CHG	RSP	0	SPT	AZM	0	FOF	MF	-1
MTZ	ATM	0	FOF	MTZ	0	NVB	CTX	-1
PEN	CHIR90	0	AMK	MIN	0	NVB	BZK	-1
TGC	MMC	0	AMK	MTZ	0	CTX	BZK	-1
AMX	CHL	0	AMK	DAP	0	BZK	ATM	-1
DOX	PIP	0	AMK	TGC	0	PRC	MEC	-1
DOX	CCCP	0	AMK	CST	0	LCX	RIF	-1

LCX	BLM	0	AMK	ASA	0	RIF	MMC	-1
AMX	RIF	0	AMK	CPO	0	CIP	CHG	-1
AMX	CCM	0	AMK	PLM	0	BAC	BZK	-1
AMX	BBR	0	CTX	MTZ	0	A22	CST	-1
CHL	FOF	0	CTX	BLM	0	DOX	AMK	0
CHL	VNL	0	BZK	ERI	0	NVB	FUS	0
CHL	MEM	0	BZK	AZM	0	CTX	IPM	0
CHL	CST	0	CFS	LOP	0	CFS	MMC	0
CHL	CCM	0	CFS	MTZ	0	ERI	PRC	0
CHL	BBR	0	CFS	PUR	0	ERI	LOP	0
CHL	PLM	0	CFS	AZM	0	CHL	PIP	0
CIP	TMP	0	CFS	BBR	0	CHL	NIT	0
CIP	CCM	0	CFS	ASA	0	DOX	NVB	0
CIP	CHIR90	0	CFS	PLM	0	CFS	RIF	0
DOX	MEM	0	ERI	MMC	0	FOF	PRC	0
DOX	ASA	0	ERI	AZM	0	FOF	CER	0
SPT	CTX	0	SMM	PRC	0	CCCP	MMC	0
FOF	SPM	0	PRC	NIT	0	MMC	BBR	0
FOF	AZM	0	PRC	RSP	0	CHL	CHG	0
FOF	BBR	0	VPM	GEN	0	DCS	RIF	0
FOF	MXF	0	LCX	BBR	0	FOF	A22	0
NVB	TMP	0	PIP	LEX	0	FOF	CHG	0
NVB	LOP	0	PIP	IPM	0	FUS	CHG	0
NVB	EGCG	0	NIT	MF	0	BZK	EGCG	0
NVB	CAF	0	NIT	AZM	0	CIP	BLM	0
NVB	PUR	0	NIT	BBR	0	LOP	MMC	0
NVB	AZM	0	NIT	GEN	0	LCX	STZ	0
NVB	BBR	0	LOP	RIF	0	CIP	CLO	0
NVB	ASA	0	LOP	IPM	0	CEC	MMC	0
NVB	MXF	0	LOP	CCM	0	ATM	MF	0
NVB	STZ	0	LOP	PLM	0	MMC	PLM	0
FUS	VNL	0	MIN	TOB	0	CHL	PEN	0
CTX	RIF	0	MIN	AZM	0	CIP	NIT	0
CTX	BBR	0	MIN	PLM	0	IPM	MEM	0
CLI	ATM	0	MIN	GEN	0	PQ	CLR	0
TMP	LCX	0	TOB	MTZ	0	IPM	CST	0
TMP	BBR	0	TOB	CLR	0	PRC	PSA	1
TMP	ASA	0	TOB	AZM	0	PSA	CST	1
TMP	MXF	0	TOB	ASA	0	CIP	PMS	-1
PQ	BBR	0	CHG	EGCG	0	DOX	PMS	-1
VNL	LCX	0	EGCG	CST	0	DOX	PYO	-1
LCX	ATM	0	RIF	AZM	0	PMS	BLM	-1
LCX	AZM	0	CLR	BBR	0	PMS	MMC	-1
LCX	CCM	0	AMK	MEC	0	PMS	CHIR90	-1

MEC	EGCG	0
MEC	BBR	0
A22	RIF	0
NSN	CHIR90	0
EGCG	MEM	0
RIF	IPM	0
RIF	MEM	0
ATM	CER	0
ATM	CPO	0
CLR	GEN	0
CAF	PUR	0
CAF	MXF	0
PUR	ASA	0
MMC	CCM	0
DOX	CAF	0
AMX	CST	0
CHL	CAF	0
PMB	CHIR90	0
FUS	CER	0
CTX	VNL	0
BZK	DIC	0
SPM	CLR	0
VNL	PUR	0
MEC	GEN	0
TGC	CHIR90	0
AMX	A22	1
DOX	LOP	1
FOF	RSP	1
FOF	ASA	1
OXA	PEN	1
BZK	PRC	1
CFS	ATM	1
CFS	CLR	1
PRC	MF	1
CCCP	LOP	1
MIN	RIF	1
CIP	MXF	1
DOX	A22	-1
FOF	FUS	-1
FOF	MIN	-1
PMB	AMK	-1
AMK	RIF	-1
BZK	CLR	-1
ERI	BBR	-1
CTX	LEX	0
CHL	CFS	0
CIP	LEX	0
CFS	LEX	0
CFS	LZD	0
CFS	DAP	0
CIP	DIC	0
CIP	TRI	0
SPT	BZK	0
SPT	VPM	0
FUS	EGCG	0
AMK	CFS	0
AMK	A22	0
CFS	TOB	0
PRC	CHG	0
PIP	MF	0
NIT	ASA	0
LEX	MMC	0
RSP	MMC	0
NON	MMC	0
PMB	DXR	0
CIP	DXR	0
DOX	PHM	0
FOF	CST	0
OXA	MMC	0
BZK	PHM	0
VNL	CHG	0
PIP	CER	0
CAF	CST	0
RSP	CST	0
CHL	TOB	0
CIP	SPM	0
CIP	TOB	0
CIP	NSN	0
CIP	PHM	0
DOX	BBR	0
DOX	GEN	0
SPT	GEN	0
AMK	TPH	0
AMK	BLM	0
BZK	LCX	0
ERI	PHM	0
NIT	A22	0
A22	LOP	0
PMS	PLM	-1
TMP	PYO	-1
LCX	PYO	-1
ATM	PYO	-1
PUR	PYO	-1
PYO	MMC	-1
PYO	PLM	-1
PYO	MXF	-1
PMS	MXF	-1
PMS	GEN	-1
NVB	PMS	-1
PMS	VNL	0
PMS	NIT	0
PMS	RIF	1
PRC	PYO	1
PMS	MEC	-1
CHL	PMS	-1
DOX	PSA	-1
PSA	CHIR90	0
CHL	PYO	-1
CLI	PYO	-1
PMS	CLI	-1
PMS	EGCG	1
AMK	PYO	-1
TOB	PYO	-1
CLR	PYO	-1
AMK	PMS	-1
PYO	AZM	-1
PMS	PQ	0
PMS	PRC	1
PMS	IPM	0
PMS	ASA	1
NIT	PYO	0
PYO	ASA	1
CIP	PYO	-1
PMS	LCX	-1
PMS	ATM	-1
PYO	BLM	-1
PYO	CHIR90	-1
PYO	GEN	-1
NVB	PYO	0
PMS	CLR	-1
PMS	AZM	-1
MEC	PYO	-1

RIF	PLM	-1	A22	MIN	0	DCS	PMS	0
CIP	RIF	-1	MIN	NSN	0	PMB	PSA	0
AMK	VNL	-1	MIN	TPH	0	PMS	PYO	0
AMK	AZM	-1	MIN	CER	0	PMS	CCM	0
SPM	TOB	-1	MIN	MMC	0	PMS	STZ	0
ERI	TOB	-1	MIN	BBR	0	BZK	PYO	0
A22	BBR	-1	CHG	PUR	0	VNL	PSA	0
SPT	LOP	0	CHG	AZM	0	VPM	PSA	0
FOF	CTX	0	CHG	BBR	0	LOP	PSA	0
FOF	NIT	0	CHG	PLM	0	CHG	PYO	0
PMB	ERI	0	ATM	PHM	0	EGCG	PYO	0
AMK	CTX	0	CLR	AZM	0	PYO	IPM	0
CTX	PIP	0	PUR	CER	0	PYO	CCM	0
SMM	MMC	0	MMC	CST	0	PYO	BBR	0
A22	ATM	0	MMC	PHM	0	PYO	DIC	0
CLO	MMC	0	A22	EGCG	0	PSA	CCM	0
BZK	LOP	0	AMX	CHG	0	PSA	BBR	0
CIP	ASA	0	CHL	AMK	0	PSA	ASA	0
FOF	PUR	0	OXA	CHG	0	PMS	MEM	0
CTX	ASA	0	PMB	CTX	0	PYO	MEM	0
PRC	A22	0	AMK	NSN	0	SPT	PSA	0
PRC	MEM	0	BZK	PEN	0	PMS	PIP	0
NIT	CHIR90	0	BZK	LZD	0	PMS	PEN	0
RIF	PUR	0	BZK	STZ	0	PIP	PYO	0
ATM	PUR	0	CFS	NSN	0	PEN	PYO	0
ATM	CST	0	CHG	BLM	0	PYO	PSA	0
CLR	ASA	0	TPH	IPM	0	PSA	STZ	0
BZK	CCM	0	CIP	STZ	0	PMS	RSP	0
TOB	RIF	0	DCS	PQ	0	CCCP	PSA	0
AMX	DCS	1	DCS	MEC	0	CHG	PSA	0
AMX	FOF	1	DCS	CLR	0	PYO	STZ	0
AMX	CEC	1	DCS	IPM	0	ERI	PYO	0
AMX	MEC	1	DOX	SPT	0	AMX	PMS	0
AMX	ATM	1	DOX	BZK	0	PMS	MIN	0
DCS	ASA	1	DOX	CST	0	CCCP	PYO	0
DOX	VPM	1	FOF	OXA	0	CFS	PYO	1
SPT	VNL	1	FOF	MEC	0	AMX	PYO	-1
FOF	CFS	1	FOF	NSN	0	SPT	PYO	-1
FOF	VNL	1	FOF	IPM	0	CTX	PMS	-1
FOF	PEN	1	BAC	PEN	0	CTX	PYO	-1
NVB	BAC	1	FUS	MF	0	PMS	TMP	-1
NVB	PMB	1	FUS	AZM	0	PMS	A22	-1
BAC	TMP	1	AMK	PQ	0	PMS	PUR	-1
BAC	MEC	1	AMK	MXF	0	PMS	PSA	-1

BAC	IPM	1	AMK	STZ	0	PMS	BBR	-1
PMB	FUS	1	BZK	MTZ	0	A22	PYO	-1
AMK	PUR	1	CEC	PIP	0	PMS	ERI	-1
CTX	MEC	1	PQ	STZ	0	PSA	IPM	-1
CTX	MEM	1	PRC	PIP	0	PMS	CEC	0
CEC	MEC	1	PRC	CCM	0	PMS	CCCP	0
CEC	A22	1	PRC	ASA	0	PMS	TPH	0
CEC	ATM	1	PRC	CPO	0	PMS	DXR	0
CFS	TMP	1	PRC	MXF	0	CEC	PYO	0
CFS	MEM	1	VNL	RIF	0	CLO	PYO	0
TMP	SMM	1	VNL	CCM	0	RSP	PSA	0
PRC	RIF	1	VNL	STZ	0	SPT	PMS	0
PRC	AZM	1	VPM	PEN	0	FOF	PYO	0
PRC	PLM	1	PIP	MEC	0	FOF	PSA	0
VNL	EGCG	1	NIT	STZ	0	NVB	PSA	0
VPM	CCCP	1	CHG	RIF	0	VNL	PYO	0
NIT	PLM	1	EGCG	RIF	0	PSA	MF	0
CCCP	CST	1	EGCG	ASA	0	PMS	CFS	1
CAF	ASA	1	RIF	DXR	0	NIT	MIN	0
BLM	CST	1	RIF	CER	1	5FC	MMC	0
BLM	STZ	1	RIF	STZ	0	VNL	MIN	0
CER	CST	1	ATM	STZ	0	CST	PHM	0
CHL	CHIR90	1	CER	STZ	0	AMK	5FC	0
BAC	CHIR90	1	FOF	CEC	0	CTX	5FC	0
FUS	CHIR90	1	BAC	ATM	0	PIP	5FC	0
VPM	CHIR90	1	CTX	CHIR90	0	NIT	5FC	0
A22	MEM	1	CEC	IPM	0	TOB	5FC	0
CLR	CHIR90	1	PQ	TOB	0	CHG	5FC	0
CHL	CER	-1	PQ	MEM	0	5FC	ATM	0
CIP	VNL	-1	VPM	MEC	0	5FC	CST	0
DCS	BZK	-1	MEC	ATM	0	5FC	CCM	0
DOX	FOF	-1	MEC	PEN	0	ATM	IPM	0
DOX	TMP	-1	DXR	MXF	0	MEM	CPO	0
DOX	VNL	-1	AMX	MMC	0	MEM	PLM	0
DOX	MEC	-1	CHL	LCX	0	CST	GEN	0
DOX	IPM	-1	CHL	CLR	0	5FC	MEM	0
DOX	CER	-1	MIN	5FC	0	CST	CPO	0
SPT	ERI	-1	PMB	5FC	0			

Note: 1, 0 and -1 represent synergistic, additive and antagonistic drug combination, respectively.
